# Long-term impact of adult WHO grade II or III gliomas on health-related quality of life: A systematic review

**DOI:** 10.1093/nop/npab062

**Published:** 2021-11-10

**Authors:** Sé Maria Frances, Galina Velikova, Martin Klein, Susan C Short, Louise Murray, Judy M Wright, Florien Boele

**Affiliations:** Leeds Institute of Medical Research at St James’s, St James’s University Hospital, University of Leeds, Leeds, UK; Leeds Institute of Medical Research at St James’s, St James’s University Hospital, University of Leeds, Leeds, UK; Leeds Institute of Molecular Research, University of Leeds, Leeds, UK; Department of Clinical Oncology, Leeds Cancer Centre, Leeds, UK; Department of Medical Psychology, Cancer Center Amsterdam, Brain Tumor Center Amsterdam, Amsterdam UMC, Vrije Universiteit Amsterdam, Amsterdam, the Netherlands; Leeds Institute of Medical Research at St James’s, St James’s University Hospital, University of Leeds, Leeds, UK; Leeds Institute of Health Sciences, Faculty of Medicine and Health, University of Leeds, Leeds, UK; Leeds Institute of Molecular Research, University of Leeds, Leeds, UK; Department of Clinical Oncology, Leeds Cancer Centre, Leeds, UK; Leeds Institute of Molecular Research, University of Leeds, Leeds, UK; Department of Clinical Oncology, Leeds Cancer Centre, Leeds, UK; Academic Unit of Health Economics, Leeds Institute of Health Sciences, University of Leeds, Leeds, UK; Leeds Institute of Medical Research at St James’s, St James’s University Hospital, University of Leeds, Leeds, UK; Leeds Institute of Health Sciences, Faculty of Medicine and Health, University of Leeds, Leeds, UK

**Keywords:** adult, glioma, health-related quality of life, long-term, survivorship

## Abstract

**Background:**

Glioma diagnosis can be devastating and result in a range of symptoms. Relatively little is known about the long-term health-related quality of life (HRQOL) challenges faced by these patients. Establishing the impact of diagnosis on HRQOL could help positively tailor clinical decision making regarding patient support and treatment. The aim of this review is to identify the long-term HRQOL issues reported at least 2 years following diagnosis of WHO grade II/III glioma.

**Method:**

Systematic literature searches were carried out using Medline, EMBASE, CINAHL, PsycINFO, and Web of Science Core Collection. Searches were designed to identify patient self-reports on HRQOL aspects defined as physical, mental, or social issues. Quality assessment was conducted using the Mixed Methods Appraisal Tool (MMAT). Narrative synthesis was used to collate findings.

**Results:**

The search returned 8923 articles. Two hundred seventy-eight titles remained after title and abstract screening, with 21 full-text articles included in the final analysis. The majority of studies used quantitative methods, with 3 articles reporting mixed methodology. Negative emotional/psychological/cognitive changes were the most commonly reported. Physical complaints included fatigue, seizures, and restricted daily activity. Social challenges included strained social relationships and financial problems. Patient coping strategies were suggested to influence patient’s survival quality.

**Conclusion:**

The consequences of a glioma diagnosis and treatment can have substantial implications for patients’ long-term HRQOL and daily functioning. Findings from this review lay the groundwork for efforts to improve patient HRQOL in long-term survivorship.

Gliomas represent 78% of primary malignant brain tumours.^1^ Of these tumours, Oligodendrogliomas and Astrocytomas represent 4.5% ad 16.7% of these respectively.^2^ Gliomas and their treatment can result in noticeably impaired health-related quality of life (HRQOL).^3^ Patients often experience fatigue, cognitive deficits, and mood disturbances.^4^ The chosen treatment and prognosis of glioma depends largely on tumor histology and molecular profile.^5^

While still burdensome, World Health Organisation (WHO) grade I brain tumors typically have a good prognosis.^2^ WHO grade IV brain tumors represent the other end of this spectrum of malignancy, with patients usually experiencing rapid disease progression and tumor recurrence. In this article, we will focus exclusively on WHO grade II or III gliomas, which are diffuse and malignant gliomas with intermediate prognosis. Survival ranges from 4 to 16 years following initial diagnosis,^6^ and patients receive multimodal treatments primarily with the aim of delaying disease progression and extending survival. Given this prognosis, consideration of HRQOL in long-term survival is of increasing importance in both clinical and social care settings for patients with WHO grade II and III gliomas.

Patients experience significant life changes immediately following diagnosis, such as the introduction of treatment and management of symptoms, changes in daily activities and alterations to their social support system.^7^ However, less is known about the longer-term experiences of these patients as they attempt to return to their day-to-day lives. Given that prognosis varies greatly between brain tumor groups, there is no clear or universal definition of “long-term survival” in neuro-oncology. Here, we define “long-term survival” in WHO grade II and III gliomas as ≥2 years since diagnosis. A period of this length following diagnosis will have allowed patients to adjust to living with their diagnosis, and patients will have completed first-line treatment with potential late effects now starting to emerge. We anticipate that the long-term impact of the disease and treatment on their HRQOL will be clear from ≥2 years after diagnosis. We also expect that overall, HRQOL impairments in long-term survival will be milder than in the acute phase, as there will have been physical and emotional recovery and adjustment.^8^

Despite a growing interest in HRQOL within the field of oncology, the evidence-base within rarer malignancies such as glioma lags behind more prevalent patient groups such as breast or lung. Similarly, within neuro-oncology, grade II and III gliomas are relatively rare compared to the more common tumors, for example, glioblastoma (GBM). As a result, literature on long-term HRQOL in grade II and III gliomas exclusively is limited. Furthermore, existing literature in neuro-oncology commonly divides gliomas into low-grade (WHO grade I and II) and high-grade (WHO grade III and IV). These subgroups are becoming less relevant after the WHO 2016 tumor reclassification,^9^ which places greater emphasis on tumor behavior. Yet, this can complicate assessing HRQOL in survivorship of WHO grade II and III gliomas. To our knowledge, there has been no systematic review collating evidence to provide an overview of the long-term HRQOL issues faced by WHO grade II/III glioma patients.

Therefore, we performed a systematic review of quantitative, qualitative, and mixed-methods evidence, to present an overview of HRQOL in survivors of grade II/III glioma. By identifying common experiences from patient self-reported HRQOL, this review will offer new insights into the impact of diagnosis and/or treatment on long-term survival. Through enhancing our understanding of these impacts, this review could be invaluable in tailoring clinical decision making to improve HRQOL in patients with grade II or III glioma.

## Methods

### Search Methods

The following databases were searched: Medline (Ovid), Embase (Ovid), PsycINFO (Ovid), and PubMed and Web of Science Core Collection. Gray literature such as conference abstracts and theses were identified in Embase, PsycINFO, and Web of Science. These searches were completed on June 26, 2020 and updated on July 29, 2021. The search terms and strategies were created with advice from an information specialist, specifically for the following concepts: brain tumors, adults, quality of life, and long-term survivorship. Search strategies were developed using a combination of free-text terms and subject headings. Searches were limited to literature published in English. No limit was placed on time since publication. See Supplementary material 1 for the complete search strategy. The protocol for this review was registered on PROSPERO (CRD42020207211). Literature titles found were exported to EndNote X9 software where the duplicate removal function was used, and title screening was carried out.

### Selection Criteria

Primary, peer-reviewed, and gray literature was included according to the following criteria:

1. Human, adult participants (≥18 years old);
2. Diagnosis of a primary brain tumor/glioma;
3. Tumor pathology must be a histologically confirmed WHO grade II or III glioma. If the study had a mixed participant group, then reports were included if the majority of participants were eligible (≥50% WHO grade II/III);
4. Mean/median time since diagnosis (TSD) must have been ≥2 years. This cutoff allowed us to assess HRQOL after initial treatment(s), when patients start to resume their normal lives—hence providing the earliest indication of “long-term survival”;
5. English language/translation.

Exclusion criteria were as follows:

1. Articles not published in English;
2. Reviews, case studies, and case reports;
3. Reporting on WHO grade I, IV, or brain metastases/secondary brain tumors;
4. Studies using only non-self-reported measures of HRQOL, for example, performance outcomes or clinician- or proxy-reported outcomes.

In 2 stages (title/abstract and full text), articles were assessed for eligibility by the lead investigator (S.F.). A second reviewer (F.B.) independently screened a random sample (20%) at each stage. Of these original libraries, we found a discrepancy of 14%. Discrepancies were discussed, and the lead reviewer (S.F.) revisited hits to ensure consistency in study selection.

### Data Extraction and Quality Assessment

Data extraction was carried out using a standardized template. Data extracted included study design, study outcomes, sample size, and participant selection criteria, as well as the selected method used to report on HRQOL. Data were extracted in line with the themes derived from Hay and Reeve^10^ definition of HRQOL—“how well a person functions in their life and his or her perceived wellbeing in physical, mental & social domains of health.” Subcategories of HRQOL were added as appropriate (eg, fatigue, emotional/psychological/cognitive functioning, coping), if necessary guided by domain definitions.^11^ We used the Mixed Methods Appraisal Tool (MMAT) for quality assessment of included studies. This tool has been validated for use in reviews with mixed methods.^12^ Following quality assessment, no studies were removed; however, studies of lower quality should be interpreted with caution and in consideration of their limitations. See Supplementary material 2 for MMAT scores.

### Narrative Synthesis

**Figure 1.**
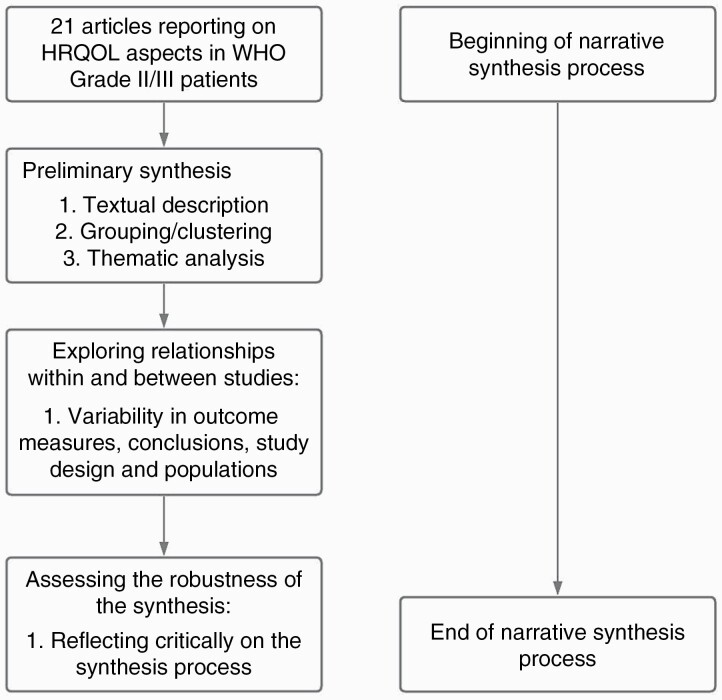
Flow chart of review process.

Narrative synthesis methodology was used to collate and interpret study findings. This type of synthesis was the most appropriate for this review due to the multiple methodology types and the variety of findings included. Figure 1 shows the process of narrative synthesis. Evidence was organised based on the themes derived from our chosen definition of HRQOL,^10^ namely physical, mental and social aspects. We also created sub-categories where appropriate e.g. fatigue, coping, positive changes.

## Results

### Search Results

**Figure 2.**
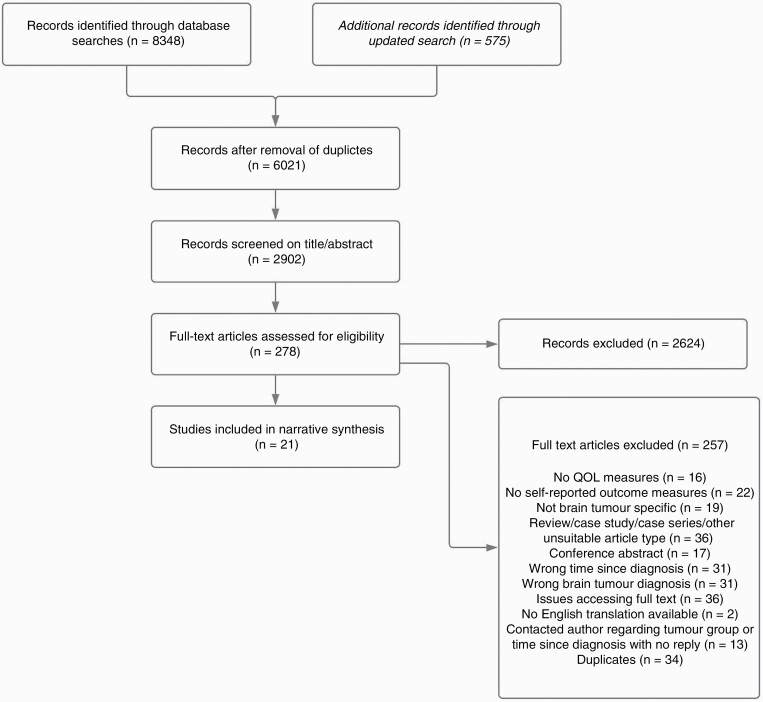
Flow chart of search process.

The findings of this review were reported in accordance with PRISMA guidelines.^13^ In total, 8923 articles were returned. Upon removing duplicates, this left 2902 titles. Two thousand six hundred twenty-four articles were excluded based on title/abstract screening, as they did not meet the inclusion criteria. Two hundred seventy-eight articles were assessed in full for eligibility, removing 235 articles that did not meet the inclusion criteria. Forty-three articles appeared eligible for inclusion. However 8 were excluded after a full-text review. Another 14 lacked details needed to be checked against the inclusion/exclusion criteria. These were excluded after contacting the corresponding authors, see Supplementary material 3. In total, 21 papers were included in the narrative synthesis. See Figure 2 for search results.

### Study Characteristics

Sample sizes for the included studies ranged from 14^14^ to 477.^15^ Most of the studies published were from western countries, with over 50% of these studies originating from Europe. Included studies used a variety of study designs (cross-sectional, n = 12; randomized controlled trial, n = 3; cohort, n = 2; pilot study, n = 1; and longitudinal, n = 3). The majority of included studies (83%) had quantitative data, with 3 papers (17%) using mixed methodologies. Five studies used comparisons to either healthy or non-brain tumor control groups, with 5 of the remaining studies drawing direct comparisons between 2 brain tumor cohorts in treatment studies (24%). Studies reported on a variety of outcome measures. See Table 1 for study characteristics and clinical cutoffs available.

**Table 1.** Study Char acteristics

Reference Number	TSD Category	Title	Year	Author	TSD (years)	Location	Sample style (n = )	Molecular Markers	Age at diagnosis	Controls/Comparison Sample	Study Aims	Instrument	Clinical Cutoff
^14^	2-5 years	Assessment of quality of life in patients treated for low-grade glioma: a preliminary report	1992	Taphoorn, Heimans, Snoek, Lindeboom, Oosterink, Wolbers Karim	2 years	the Netherlands	Adult low-grade glioma patients, treated with surgery and radiotherapy at least 1 year previously (n = 14)	Not reported	Not reported	N/A	Evaluate the utility of the instruments and assess quality of life	Affect Balance Scale (ABS) Profile of Mood States (POMS) Interview	N/A ≥7 ^52^
16	2-5 years	Cognitive functions and quality of life in patients with low-grade glioma—the impact of radiotherapy	1994	Taphoorn, Klein Schiphorst, Snoek Lindeboom, Wolbers , Karim, Huilgens, Heimans	3.5 years	the Netherlands	Adult patients >18 years with low-grade supratentorial glioma without clinical or CT signs of recurrence (+ or − surgery and radiotherapy at least 1 year previously	Not reported	Not reported	Patients treated with radiotherapy (LGG/RT+) vs those without (LGG/RT−) Control—Patients with non-Hodgkin’s lymphoma, chronic lymphatic leukemia (NHL/CLL)	Do the severe disturbances observed in LGG patients are related to the tumor, the radiotherapy, or the malignancy caused psychological problems	Profile of Mood States (POMS)	7 ^52^
^19^	2-5 years	Health-related quality of life in patients treated for anaplastic oligodendroglioma with adjuvant chemotherapy: results of an EORTC randomised controlled trial	2007	Taphoorn, van den Bent, Mauer, Coens, Delattre, Brandes, Smitt, Bernsen, Frenay, Tijssen, Lacombe, Allgeier, Bottomley	2 years+	Multi-country	Anaplastic oligodendroglioma, treated with RT+/− adjuvant chemotherapy (combined Procarbazine, Lomustine, Vincristine, n = 50)	Not reported	Not reported	RT+ vs RT− adjuvant chemotherapy	Impact of adjuvant chemotherapy on HRQOL	EORTC QLQ-C30 EORTC QLQ-BN20	90 (functioning domains) ^53^ 5-10 (symptom domains) ^53^ N/A
33	2-5 years	Surgical strategies in low-grade gliomas and implications for long-term quality of life	2014	Jakola, Unsgard, Myrmel, Kloster, Torp, Sagberg, Lindal, Solheim	≥2 years	Sweden	18 years, diagnosis of grade I or II low-grade glioma, biopsy and watchful waiting; early resection (n = 153)	Not reported	Not reported	Comparison of those that favored biopsy vs resection	Compare the long-term HRQOL in 2 hospital cohorts with different surgical strategies	Euro-Qol 5D (EQ-5D) EORTC QLQ-C30 EORTC QLQ-BN20	0.10 ^54^ 90 (functioning domains) ^53^ 5-10 (symptom domains) ^53^ N/A
17	2-5 years	Factors associated with health-related quality of life in patients with glioma: impact of symptoms and implications for rehab	2020	Umezaki, Shinoda, Mukasa, Tanaka, Takayanagi, Oka, Tagawa, Haga Yashino	2.9 years	Japan	WHO grade II-IV glioma (n = 66)	Grade II Diffuse astrocytoma (IDH-mutant/wildtype or NOS) (n = 8) IDH-mutant, 1p19q-codeleted Oligodendroglioma NOS/Oligoastrocytoma NOS (n = 7, n = 3) Diffuse glioma, IDH-wildtype, NES Grade III IDH-mutant Anaplastic astrocytoma (n = 9) IDH-wildtype Anaplastic astrocytoma. NOS (n = 2) Anaplastic oligodendroglioma (n = 7) IDH-mutant, 1p19 codeleted Anaplastic glioma, IDH-wildtype NES (n = 1)	Not reported	N/A	Investigate the impact of symptoms on quality of life—focused on social participation because many patients with glioma are of working age	EORTC QLQ C30 EORTC QLQ-BN20	90 (functioning domains) ^53^ 5-10 (symptom domains) ^53^ N/A
29	2-5 years	Emotional concerns and coping strategies in low-grade glioma patients	2017	Moreale, Campanella, Marin, Skrap, Pales	3.2 years	Italy	Surgically treated WHO low-grade glioma (n = 36)	Not reported	Not reported	Caregivers—capable of responding to a face-to-face interview	Advance knowledge in the field of depression, anxiety, and coping strategies enacted by LGG patients by measuring their prevalence in their postsurgical period. To establish whether caregivers can reliably report these concerns as surrogate informants	Beck Depression Inventory (BDI) State-Trait Anxiety Inventory (STAI) Jaloweic Coping Strategy (JCS)	0-9 indicate no or minimal depression; 10-19 indicate mild to moderate depression, etc. ^55^ 40 ^56^ N/A
^32^	2-5 years	Long-term cognitive functioning and psychological wellbeing in surgically treated patients with low-grade glioma	2017	Campanella, Palese, Del Missier, Moreale, Lus, Shallice, Fabbro, Skrap	3.35 years	Italy	Surgically treated patients with radiologically stable low-grade glioma, fluent in Italian with no sign of tumor progression (n = 50)	Not reported	Not reported	N/A	Investigation of long-term cognitive and affective functioning and psychological well-being	STAI Beck Depression Inventory (BDI)	40 ^56^ 0-9 indicate no or minimal depression; 10-19 indicate mild to moderate depression, etc. ^55^
	2-5 years	The quality of life of patients with malignant gliomas and their caregivers	2008	Muñoz Juarez, Muñoz Portno, Fineman Badie Mamela, Ferrell	2.86	United States	Age 18 or older, no tumor recurrence, or progression after initial diagnosis, life expectancy ≥3 months, KPS ≥ 70 (n = 20)	Not reported	Not reported	Caregiver ratings	What aspects of QOL disruption are reported by patients with malignant gliomas? What positive aspects of the experience of glioma are reported by patients?	FACT-Br QOLCS	N/A N/A
^30^	2-5 years	Internet-based guided self-help for glioma patients with depressive symptoms: a randomised controlled trial	2018	Boele, Klein, Verdonck-de Leeuw, Cuijpers, Heimans, Snijders, Vos, Bosma, Tijssen, Reijneveld	3.44 years	the Netherlands	Adult glioma patients, grade II, III, and IV at least mild depressive symptoms (n = 89)	Not reported	45 years (mean)	Patients with aematological malignancies	Levels of depressive symptoms by means of a low-intensity form of CBT, delivered online to increase accessibility	Center for Epidemiological Studies-Depression (CES-D) Short Form 36 (SF-36)	≥16 ^57^ N/A
^15^	2-5 years	Health-related quality of life in patients with high-risk low-grade glioma (EORTC 22033-26033): a randomised open label phase 3 intergroup study	2016	Reijneveld, Taphoorn, Coens, Bromberg, Mason, Hoang-Xuan, Ryan, Hassel, Enting, Brandes, Wick, Chinot, Reni, Kantor, Thiessen, Klein, Verger, Barchers, Hau, Bock, Smits, Galfnopoulos Garlia, Bottomley, Stupp, Baumert	5.6 months since di- agnosis to treatment—follow- up to 36 months	Multi-country —Europe (Austria, Belgium, France, Germany, Hungary, Italy, the Netherlands, Portugal, Spain, Sweden, Switzerland, and United Kingdom); Asia and Oceania (Australia, New Zealand, and Singapore); North America (Canada [NCIC group]); the Middle East (Egypt and Israel)	Adult patients 18+, WHO histologically confirmed diffuse grade II astrocytoma, oligodendroglioma, oligoastrocytoma, WHO performance status ≥2, without previous chemotherapy who needed active treatment other than surgery (n = 477)	1p status (deleted vs non-deleted vs indeterminate)	40 years (mean)	Radiotherapy vs Chemo- therapy	Determine whether temozolomide compromises HRQOL and global cognitive functioning to a lesser extent than does radiotherapy	EORTC QLQ-C30 EORTC QLQ-BN20	90 (functioning domains) ^53^ 5-10 (symptom domains) ^53^ N/A
27	2-5 years	Psychosocial functioning and quality of life in patients with primary brain tumors	1996	Weitzner, Meyers, Byrne	2.5 years	United States	Primary brain tumor patients undergoing treatment (n = 50)	Not reported	Not reporter	N/A	Evaluate the multidimensional aspects of QOL of patients with primary brain tumors	Ferrans and Powers Quality of Life Index for Cancer (FP-QLI) Psychosocial Adjustment to Illness Scale–Self Report (PAIS-SR)	N/A N/A
^22^	5-10 years	The prevalence of altered body image inpatients with primary brain tumours: an understudied population	2020	Rowe, Vera, Acquaye, Crandon, Shah, Bryla, Wu, Wall, Siegel, Reyes, Penas-Prado, Leggiero, Cordova, Burton, Antony, Boris, Aboud, Vyas, Mathen, Gilbert, Capmhausen, Mendoza, Armstrong	5 years (median)	United States	≥18 years old patients with histologically confirmed PBT, with intracranial only disease	Not reported	Not reported	N/A	Address the prevalence of body image concerns (BIS) in PBT patients using validated questionnaires and explore contributing psychological, disease, and treatment-related factors	BIS Appearance Schemas Inventory–Revised MD Anderson Symptom Inventory-Brain Tumor Module (MDASI-BT) Patient-Reported Outcomes Measurement Information System (PROMIS)	≥10 N/A N/A N/A
^25^	5-10 years	Compromised health-related quality of life in patients with low-grade glioma	2011	Aaronson, Taphoorn, Heimans, Postma, Gundy, Beute, Slotman, Klein	5.6 years	the Netherlands	Low-grade glioma patients with no clinical signs of tumor recurrence for >1 year after histologic diagnosis and primary treatment, and no radiologic signs of recurrence within 3 months before participation (n = 195)	Not reported	Not reported	Patients with non-Hodgkin’s lymphoma, chronic lymphatic leukemia (NHL/CLL) Health Controls Group—general population normative sample	Reporting on the prevalence of generic and brain cancer-specific HRQOL problems among patients with LGG	Short Form 36 (SF-36) (Dutch)	N/A
^21^	5-10 years	Self-efficacy for coping with cancer in glioma patients measured by the CBI-B	2019	Kohlmann, Janko, Ringel, Renovanz	5.7 years	Germany	Diagnosis of a glial cerebral tumor (n = 37)	Not reported	49 years (mean)	N/A	Impact of self-efficacy for coping with cancer on distress and supportive care needs	Cancer Behaviour Inventory–Brief (CBI-B) Distress Thermometer (DT)	N/A ≥3 ^58^
^20^	5-10 years	Health related quality of life in long-term survivors with grade II gliomas: the contribution of disease recurrence and KPS	2015	Okita, Narita, Miyahara, Miyaluta, Ohno, Shibiu	5.8 years	Japan	Long-term survivors of grade II gliomas (n = 80)	Not reported	33 years (median)	N/A	Relationship between HRQOL and time since treatment, KPS, history of recurrence, and radiotherapy	EORTC QLQ C30 EORTC QLQ BN20	90 (functioning domains) 5-10 (symptom domains) ^53^ N/A
^59^	5-10 years	Quality of life in brain tumor patients and their relatives heavily depends on social support factors during the covid-19 pandemic	2021	Troschel, Ahndorf, Wille, Brandt, Jost, Eich, Stummer, Wiewrodt, Jetschke, Wiewrodt	6 years	Germany	Adult brain tumor patients (n = 63)	Not reported	Not reported	Comparison to relatives	Assess QOL in brain tumor patients and their relatives across a 12-week timespan during the first COVID-19-related lockdown	HADS Distress Thermometer WHO5 well-being score	≥8 ≥3 ^58^ ≤28 ^60^
^31^	5-10 years	Long-term cognitive dysfunction after radiation therapy for gliomas	2019	Halbo-Classen, Amidi, Wu, Lukacova, von Oettingen, Gottrup, Zachariae, Høyer	7.3 years	Denmark	Adult patients with glioma or medulloblastoma (n = 110)	Confirmed tumors grades I-III according to WHO 2016 guidelines	54.9 years	Neurosurgery + RT vs neurosurgery alone	Compared cognitive functioning in brain tumor patients	Patient Assessment of Own Functioning (PAOFI)	10.78 ^61^
^34^	5-10 years	Long-term outcomes and late adverse effects of a prospective study on proton radiotherapy for patients with low-grade glioma	2019	Tibrizi, Yeap, Sherman, Nachtigal, Colvin, Dworkin, Fullerton, Daartz, Royce, Oh, Batchelor, Curry, Loeffler, Shish	6.8 years (median)	United States	LGG patients if they had an indication for radiation therapy, age >18 years, KPS score >70 (n = 120)	IDHI1-R132H mutation status was available for 17 tumors —71% carried the mutation. 1p19q co-deletion status was available for a different set of 17 tumors—29% carried this co-deletion	Not reported	N/A	Examine the long-term morbidity following proton therapy in this updated prospective cohort of patients with LGG	FACT-G FACT-Br FACT-fatigue	N/A N/A N/A
^20^	10+ years	Health-related quality of life in stable, long-term survivors of low-grade glioma	2015	Boele, Douw, Reijneveld, Robben, Taphoorn, Aaronson, Heimans, Klein	6 years (mid-term), 12 years (long-term)	the Netherlands	Histologically confirmed oligodendroglioma, astrocytoma, and oligoastrocytoma diagnosed at least 1 year before study start, clinically stable for at least 1 year (long-term, n = 67; mid-term, n = 65)	Not reported	32 years (mean)	Individually matched healthy controls	Changes in HRQOL in long-term survivors of grade I or II astrocytoma, oligodendroglioma or oligoastrocytoma, evaluate the severity of compromised HRQOL at mid and long-term assessment	Short Form 36 (SF-36) EORTC QLQ-BN20	N/A N/A
^24^	10+ years	The relationship between function, quality of life and coping in patients with low-grade glioma	2006	Gustafsson, Edvardsson, Ahlström	10 years (median)	Sweden	LGG patients aged 18 and older, living in Örebro (n = 39)	Not reported	Not reported	N/A	Describe function, quality of life, and coping with illness-related problems in patients	EORTC QLQ-C30 Ways of Coping Questionnaire	90 (functioning domains) 53 5-10 53
^18^	10+ years	Health-related quality of life and cognitive functioning in long-term anaplastic oligodendroglioma and oligoastrocytoma	2013	Habets, Taphoorn, Nederend, Klein, Delgadillo, Hoang-Xuan, Bottomley, Allgeier, Seute, Gijtenbeek, Gans, Enting, Tijssen, van den Bent, Reijneveld	12.1 years	the Netherlands	Long-term survivors of WHO grade III gliomas (n = 32)	1p/19q co-deletion and non-1p/19q deletion	Not reported	Healthy controls	Examine the long-term functioning of anaplastic glioma patients and the HRQOL and cognitive functioning	EORTC QLQ C30 EORTC QLQ BN20	90 (functioning domains) 53 5-10 (symptom domains) 53 N/A

Abbreviations: CBT, Cognitive Behaviour Therapy; CLL, Chronic Lymphatic Leukemia; CT, Computerised Tomography; FACT-Br, Functional Assessment of Cancer Therapy - Brain; FACT-fatigue, Functional Assessment of Cancer Therap - Fatigue; FACT-G, Functional Assessment of Cancer Therapy - General; IDH, Isocitrate Dehydrogenase; KPS, Karnofsky Performance Scale; LGG, Low Grade Glioma; NES, Not Elsewhere Specified; NHL, Non-Hodgkins Lymphoma; NOS, Not Otherwise Specified; PBT, Primary Brain Tumour; QOL, Quality of Life; QOLCS, Quality of Life Cancer Survivor; RT, Radiotherapy.

### Physical Functioning

Twelve articles reported issues relating to physical functioning aspects of HRQOL.^14,16–27^ All of these studies measured HRQOL quantitatively through validated outcome measures (eg, EORTC-C30, EORTC-BN20), and 2 studies also included qualitative measures.^14,22^ Apart from two Japanese studies,^17,28^ the remaining evidence was from western countries. Four of these papers compared their respective sample to controls.^16,18,20,25^ These 3 control groups were 2 “healthy population” groups, and 2 with non-CNS cancer group (diagnosed with non-Hodgkin’s lymphoma and chronic lymphatic leukemia). In these studies, glioma patients reported impaired physical functioning.^16,18,20,25^

Many of the commonly used, validated measures of HRQOL contain some assessment of physical functioning as part of their overall score. Several studies found overall physical functioning to be impaired in their sample.^20,23^ Two studies found that patients reported difficulties with motor functioning,^17,18^ with one of these studies finding significantly higher levels of impairment compared to healthy controls.^18^ Similarly, a mixed-methods study found that in an open-ended feedback measure, patients reported difficulty with mobility in the form of issues maintaining daily routine.^23^ Patients also reported other physical complaints such as epilepsy, headaches,^25^ loss of independence, hair loss, weight gain, and vision problems.^21^ These reflect some of the long-term physical issues faced by glioma patients that can influence their HRQOL.

### Fatigue

Increased levels of fatigue proved to be a common complaint,^17,19,24,28^ and one study showed fatigue to be notably worse compared to controls^16^ One study reported tiredness and sleep disturbances as affecting 50% of patients^24^ Another study found that in examining factors related to quality of life measurements, insomnia had a statistically significant effect on patient’s perceived global health status (GHS).^17^ It is important to note that of these studies reporting on fatigue, one examines the late effects of radiotherapy and adjuvant chemotherapy on HRQOL.^19^ In this case, conclusions are limited to patients undergoing those specific treatments, as opposed to general, long-term HRQOL for all glioma patients.

### Mental Functioning

#### Psychological/Emotional Functioning (EF)

Impairments to psychological and/or EF were reported in 12 papers.^14,16,17,21–24,28–34^ This was measured across various HRQOL measures that include EF as a subscale, as well as other validated scales specifically for other psychological or emotional impairment (eg, Hospital Anxiety and Depression Scale [HADS],^35^ Profile of Mood States [POMS],^36^ Positive Affect Negative Affect Scale [PANAS],^37^ Affect Balance Scale [ABS],^38^ Center for Epidemiological Studies–Depression [CES-D]^39^). Ten of these articles originated from western countries, with the remaining study occurring in Japan.^17^ Three of these studies included qualitative methods alongside their validated scale measures.^14,16,22^

Multiple studies found evidence of glioma patients’ depressive symptoms.^14,16,17,21,31,32^ These studies had reports of depression,^16,30,31^ anxiety^29,31^ as well as anger, tension,^14^ future uncertainty,^17^ impaired EF^33^ and increased levels of psychological distress^16,21–24^ and negative affect.^32^ Three studies^14,16,31^ found clinically significant levels of depressive symptoms as measured by screening instruments (ie, POMS,^36^ HADS,^35^ BDI^40^) by a small margin relative to the clinical cutoffs displayed in Table 1. Another 3 studies^21,29,32^ also found elevated scores of depressive symptoms, which did not reach clinically or statistically significant differences compared to controls. Similarly, samples did not surpass their respective clinical cutoffs. One finding of note is that patients reported high levels of both positive and negative affect, indicating higher emotional reactivity than the reference “healthy” population.^32^ Interestingly, one study assessing the quality of life of brain tumor patients in the context of the COVID-19 pandemic found that across the first nationally imposed lockdown in Germany, patients showed significant levels of distress, anxiety, and depression, with around 23% of patients reporting elevated levels of depression symptom load.^41^ Overall, studies appear to suggest that while differing across measures, glioma patients clearly endure some level of mood/emotional disturbance.

In the qualitative strands of the 3 mixed methodology studies, evidence of emotional disturbance was reported.^22,23^ Negative affect was also reported to increase in patients, with one of these studies finding that half of the patients complained of mood disturbances (56%), with a smaller percentage reporting difficulties dealing with change (26%).^22^ Another of these studies found that negative outcomes relating to psychological well-being included fear of recurrence and distress over treatment and initial diagnosis.^23^ However, this same study also found that part of this distress could be attributed to lack of information and support from medical staff, particularly in regards to coping with their cancer diagnosis.^23^

### Coping Styles

Patients’ self-efficacy for coping with cancer (SECC) also influenced their chosen coping strategy, and determined how heavily they relied on external sources of support. Subsequently, this study found that patients with greater SECC reported lower unmet psychological needs.^21^ Interestingly this article also found that patients with greater SECC reported lower unmet needs in regards to their respective health care services, the amount of information provided as well as the support and patient care.^21^ This aligns with the findings described above, suggesting the link between patient distress and lack of information and support provided by medical staff.^23^

One study found that, patients under-utilized the coping strategies available to them^29^ with confrontative and optimistic styles reported as the most frequently used. In this study, there was only evidence of depression in 13% of the sample (full sample, n = 46), in line with previous findings in brain tumor patients.^42^ These findings could suggest that the chosen coping strategy could have significant impact on patient well-being. However, the aim of this study was to examine the reliability of caregiver ratings of emotional concerns and coping strategies, therefore we are unable to conclude any link between the effectiveness of these different coping styles alongside ratings of depression.

### Positive Change

While there are many negative effects of glioma diagnosis on HRQOL, patients can also experience positive changes in outlook because of their diagnosis. Evidence in this review suggests that greater acceptance of change, increased perception of hope and greater sense of importance can also be a consequence of diagnosis.^22^ One study found that despite experiencing increased negative affect, that patients were satisfied with their lives overall, and perceived greater maturity and greater sense of self.^32^

#### Self-reported cognitive functioning (CF)

Impaired cognitive function is a common concern in glioma patients, and an important aspect of HRQOL. We selected studies based on their use of self-reported measures; therefore, results from studies only reporting on objectively measured CF (using performance outcomes) were not included. In this literature sample, CF was often a subscale of the utilized HRQOL measures. However, it was also a common complaint reported in the qualitative strands of the mixed-method studies.^14,16,17,18,28,33,23^

Reports of impaired CF included communication difficulties^17^ impairment of memory and problems with concentration.^16^ One qualitative report from a mixed-methods study reported the frustration felt as a result of impaired communication—1 patient described how they had “words in my head but I can’t get them out,” subsequently making daily communication difficult.^23^ Findings suggest the degree to which glioma patients might experience impaired CF could be dependent on the tumor pathology, treatment strategy as well as whether there is tumor recurrence.^28,33^ For example, one quantitative study found that there was a larger number of reports of impairment to memory, cognition, and intellectual functions in patients having undergone radiation therapy alongside neurosurgery than those who had undergone neurosurgery alone.^31^ Overall, the present review found that glioma patients’ self-reported cognitive issues are of considerable importance across their long-term survival.

#### Social functioning (SF)

Changes to lifestyle and social relationships were reported by 5 articles.^14,18,22,23,24^ This includes issues related to work and finance. Several articles reported patients experiencing financial difficulties following diagnosis^14,17,18^ and frustration with resultant impaired ability to work.^23^ Given the inclusion criteria for this review ≥18 years, we can assume that a high proportion of the samples included were of working age, so disruptions to working life could be significantly impactful. Patients’ social relationships can also suffer because of glioma diagnosis.^22,24,33^ A mixed-methods study described that altered body image concerns as a result of a glioma or treatment can put strain on both existing social relationships as well act as a hindrance to meeting new people.^22^ Two articles reported that strained personal and familial relationships could have a notable negative impact on patient HRQOL.^23,24^ Findings also suggested that positive outcomes of HRQOL were associated with greater levels of communication, support, and acceptance from family members.^22,23^ Indeed, during the COVID-19 pandemic lockdown, the number of social interactions per week was associated positively with patient HRQOL, demonstrating the interdependent relationship between psychological, emotional, and social functioning.^41^

## Discussion

Despite HRQOL and long-term survivorship research becoming a prominent part of oncology research over the past several decades, our searches only returned 21 studies. The reported issues that patients face were mapped across the domains outlined by the WHO definition of HRQOL—those of “physical, mental and social wellbeing.” ^10^ This broad definition allowed us to report on a wide variety of issues. We collated evidence from both quantitative and mixed-methods studies. The rich data available suggests there are various links between these domains, all of which contribute to patients’ overall reported HRQOL. By employing few exclusion criteria, we ensured that the full breadth of available evidence could be included and assessed.

During long-term survival, WHO grade II/III glioma patients experience a variety of physical impairments. These include issues with motor functioning, pain, and changes in appearance. These were most commonly reported in studies with samples categorized as “2-5 years since diagnosis” suggesting that physical impairments are more marked in the earlier phases of long-term survivorship. This suggests that support aimed at improving physical functioning or helping patients adjust to physical changes is best offered earlier in survivorship. Fatigue was also a frequent complaint. This is in line with previous findings—van Coevorden-van Loon et al found fatigue to be a prevalent side effect of treatment in low-grade glioma patients.^43^ Peters et al examined the impact of fatigue and HRQOL on survival in patients with high-grade glioma (WHO grade III/IV).^44^ This paper was not included in our review due to the high proportion of grade IV tumors included. However, their findings suggest that the greater the number of symptoms of fatigue, the poorer the quality of patients’ survival. Our findings confirm that WHO grade II/III patients also experience the debilitating effect of fatigue, even years after diagnosis.

Given the nature of the disease and treatment strategies employed, it is perhaps unsurprising that impaired physical functioning is a relevant factor in HRQOL of these patients. Still, the evidence suggests that more prevalent and persistent issues were found in the domains of psychological/emotional and cognitive functioning. Psychological, emotional, and cognitive functioning issues were the most common complaints reported and were reported across all 3 survivorship groups (2-5; 5-10; 10+ years), suggesting that the emotional burden experienced by patient persists across long-term survivorship. Investigations covered changes in body image perception,^22^ prevalence of depression and/or anxiety symptoms,^16,17,29–31^ declines in mental well-being,^21,32^ and increased levels of cognitive and communication difficulties.^23^ The severity of these issues varied across studies due to the different constructs measured with the various outcome measures, as well as differences in clinical cutoffs. In this review, we did not aim to cover cognitive performance outcomes, thus we excluded studies that only reported results of neurocognitive tests. We focused on including studies that used self-reported CF. This arguably provides greater insight into patient experience of cognitive functioning and HRQOL as the correlations between HRQOL and neurocognitive functioning are not straightforward.^45^

We defined long-term survival as ≥2 years since diagnosis as this period would have allowed patients to adapt to their diagnosis and to return to some version of their “normal” lives. Adaptation and coping impacts upon HRQOL with 2 studies^12,31^ suggesting that severity of emotional difficulty faced by patients could be heavily dependent on their employed coping style. This is in line with Nipp et al, which found that in a study of lung cancer patients, coping strategies and emotional support correlated with mood and ratings of HRQOL.^46^ Those that utilized acceptance coping styles reported better HRQOL and mood than those that employed more styles of self-blame and denial. Hack and Degner also found that in breast cancer patients, their long-term psychological adjustment depended on their employed coping strategy.^47^ While these samples are not directly comparable for this review, they provide important perspectives in the wider context of the experience of long-term survival in cancer patients. It may be useful to establish the importance of coping styles in glioma patients, particularly given the added cognitive difficulties they experience alongside their cancer-related disease burden. There were also reports of changes in perspective to adopt a more positive outlook and appreciation for life following diagnosis.^22^ One mixed-methods study suggested a positive correlation between spiritual well-being and HRQOL outcomes.^23^ This demonstrates the complexity of patients’ response to their diagnosis and/or treatment, and highlights an important area, which should be investigated further using qualitative study designs.

Social relationships and functioning were highlighted as important aspects of HRQOL across all 3 survival time categories. Studies reported lifestyle changes, difficulty forming^22^ and maintaining social relationships^23^ It is clear that maintaining positive social relationships is important for patient HRQOL^46,48^ yet impaired SF as measured with European Organisation for Research into Treatment of Cancer Quality of Life Questionnaire–C30 (EORTC QLQ-C30), Short Form 36 (SF-36) was not highly prevalent in this review. Although this was not a direct aim of this review, our findings are in line with previous literature, which highlights that a glioma diagnosis can carry a significant burden not just for patients, but also for their family and friends.^49^

### Strengths and Limitations

This systematic review is, to our knowledge, the first to look at long-term survival and HRQOL in WHO grade II/III glioma patients. We employed few exclusion criteria in terms of study design or methodology, which allowed us to capture the full breadth of existing evidence. We looked exclusively at patient self-reported outcomes of HRQOL, allowing direct insight into patient experience of long-term survivorship. By including evidence from mixed-methods studies, we could identify similar HRQOL themes regardless of methods used, including those HRQOL aspects that are not typically captured with validated questionnaires. The context provided by qualitative dimensions of the mixed-methods studies are particularly useful and may highlight potential avenues where interventions could be offered to patients.

However, this review has some limitations. Firstly, because there is no universal definition of “long-term” survival in neuro-oncology, we chose a pragmatic cutoff of ≥2 years after diagnosis (mean or median) as from a clinical viewpoint, these patients will have completed treatment and entered a period of follow-up. Included studies reported on mixed samples, and not all potentially eligible studies included information on TSD (see Supplementary material 3). Subsequently, we may have excluded potentially relevant studies based on this lack of information. Furthermore, by using the mean/median average TSD, studies could include patients who were assessed <2 years after diagnosis. This means that some of the included studies’ results may include patient experiences earlier after diagnosis, so conclusions for long-term quality of life should be interpreted with caution. We acknowledge that many studies included in this review recruited participants before the 2016 WHO tumor reclassification, which is likely to have influenced the samples included before and after this point, making it more difficult to draw conclusions on HRQOL for WHO grade II/III glioma. Our decision to include literature if the sample had at least 50% grade II/III tumors, may have introduced some level of bias for studies with greatly mixed samples. Thirdly, the inclusion of only English published/translation of papers could account for the lack of diversity in the locations of included studies, introducing a potential for cultural bias to our findings. The studies included also used a wide range of outcome measures (eg, EORTC QLQ-C30, HADS,^35^ POMS,^36^ etc.). Some of these are better validated within the glioma population or have clearer cutoff scores for clinical relevance than others. Therefore, it was not possible to add information on statistical as well as clinical relevance for all HRQOL outcomes in this review. Due to the nature of the samples included, we were unable to infer the effect of the different treatments used in the various tumor groups, and the influence this might have had on the HRQOL outcomes. Finally, the MMAT quality assessment indicates the quality of the studies varied, emphasizing conclusions should be assumed with caution—see Supplementary material 2.

### Future Research

In future studies, consideration should be given to the importance of coping mechanisms, self-efficacy, and resilience in managing the quality of patients’ survival. This could help inform clinical practice and allow tailoring of support services for long-term survivors of glioma. A previous review looked at identifying supportive care interventions to improve HRQOL in brain tumor patients.^50^ Across 10 randomized controlled trials included in said review, only 2 interventions were found to improve HRQOL (home-based psychosocial interventions and acupuncture with rehabilitation). Further evidence is needed to confirm the effectiveness of the current support provided to patients. This could provide the basis for further intervention studies. Particularly, as our review findings suggest that even years after diagnosis, glioma patients experience HRQOL issues. Observational studies should include age at diagnosis as well as details on treatments received to allow investigation of these factors on long-term HRQOL in WHO grade II/III glioma patients. While the studies included originated from a variety of different countries (eg, Italy, United States, Sweden, Denmark, Japan, France, the Netherlands, Germany, and Norway, etc.), there was a significant lack of studies included from non-westernized countries. This could have introduced a level of cultural bias to this review due to variations in perceptions of health and well-being^51^ as well as financial affluence across cultures. This also applies to the potential impact of spirituality and religion. Future studies could explore the influence of wealth, culture, spirituality, and religion on glioma survivors’ HRQOL using qualitative methods.

## Conclusions

This systematic review presents an up-to-date overview of the state of the evidence around HRQOL in glioma survivorship—a population confronted not just with the diagnosis and treatment of cancer, but also with a neurological condition. While physical and social issues can persist for years after diagnosis, the impact on mental functioning (psychological, emotional, and cognitive) is much more prominent. Evidence suggests that patients’ capacity to cope with long-term survivorship issues could be indicative of HRQOL, however further research is needed to establish any causal link. Findings from this review aid understanding of the impact that glioma diagnosis and treatment can have on patient HRQOL. This could help to facilitate the development of interventions aimed at improving glioma patients’ quality of survival, and help in streamlining existing resources and support for patients in clinical practice.

## Supplementary Material

npab062_suppl_Supplementary_Materials_S1

npab062_suppl_Supplementary_Materials_S2

npab062_suppl_Supplementary_Materials_S3
