# Supplementary material for: Long-term impact of adult WHO grade II or III gliomas on health-related quality of life: A systematic review
Source: Neurooncol Pract. 2021 Nov 10;9(1):3–17. doi: 10.1093/nop/npab062 (PMC8789291; doi:10.1093/nop/npab062)
Supplement: npab062_suppl_Supplementary_Materials_S1 [file npab062_suppl_supplementary_materials_s1.docx]

| 1. exp Cognition/ |  |
| --- | --- |
| 2. cognitive function.mp. |  |
| 3. (QOL or "quality of life").mp. |  |
| 4. (HRQOL or HR-QOL or "health related quality of life" or "health-related quality of life").mp. |  |
| 5. "Quality of Life"/ |  |
| 6. psychological distress.mp. |  |
| 7. emotional distress.mp. |  |
| 8. anxiety.mp. |  |
| 9. depression.mp. |  |
| 10. cognitive deficit.mp. |  |
| 11. social functioning.mp. |  |
| 12. 1 or 2 or 3 or 4 or 5 or 6 or 7 or 8 or 9 or 10 or 11 |  |
| 13. meningioma.mp. |  |
| 14. (grade 1 or grade I or grade one).ti,ab,kw. |  |
| 15. glioma*.mp. |  |
| 16. 14 and 15 |  |
| 17. 13 or 16 |  |
| 18. 17 and 12 |  |
| 19. comment/ |  |
| 20. letter/ |  |
| 21. editorial/ |  |
| 22. note/ |  |
| 23. news/ |  |
| 24. newspaper article/ |  |
| 25. meta-analysis/ |  |
| 26. Review Literature as topic/ or systematic review/ or review/ |  |
| 27. 19 or 20 or 21 or 22 or 23 or 24 or 25 or 26 |  |
| 28. (exp Child/ or Adolescent/ or Infant/) not exp Adult/ |  |
| 29. exp animals/ not exp humans/ |  |
| 30. 28 and 29 |  |
| 31. 18 not 27 |  |
| 32. 31 not 3 |  |

Search Strategy
