# Supplementary material for: Long-term impact of adult WHO grade II or III gliomas on health-related quality of life: A systematic review
Source: Neurooncol Pract. 2021 Nov 10;9(1):3–17. doi: 10.1093/nop/npab062 (PMC8789291; doi:10.1093/nop/npab062)
Supplement: npab062_suppl_Supplementary_Materials_S2 [file npab062_suppl_supplementary_materials_s2.docx]

| Reference Number  ***Supplementary Material 2 – MMAT Scores*** | Title | Year | Author | MMAT SCORE |
| --- | --- | --- | --- | --- |
| [14] | Assessment of quality of life in patients treated for low-grade glioma: a preliminary report | 1992 | Taphoorn, Heimans, Snoek, Lindeboom, Oosterink, Wolbers & Karim | 100 |
| [16] | Cognitive functions and quality of life in patients with low-grade glioma – the impact of radiotherapy | 1994 | Taphoorn, Klein Schiphorst , Snoek Lindeboom , Wolbers , Karim, Huilgens & Heimans | 75 |
| [19] | Health-related quality of life in patients treated for anaplastic oligodendroglioma with adjuvant chemotherapy: results of an EORTC randomised controlled trial | 2007 | Taphoorn, van den Bent, Mauer, Coens, Delattre, Brandes, Smitt, Bernsen, Frenay, Tijssen, Lacombe, Allgeier & Bottomley | 25 |
| [33] | Surgical strategies in low-grade gliomas and implications for long-term quality of life | 2014 | Jakola, Unsgard, Myrmel, Kloster, Torp, Sagberg, Lindal, Solheim | 100 |
| [17] | Factors associated with health related quality of life in patients with glioma: impact of symptoms and implications for rehab | 2020 | Umezaki, Shinoda, Mukasa, Tanaka, Takayanagi, Oka, Tagawa, Haga & Yashino | 100 |
| [29] | Emotional concerns and coping strategies in low-grade glioma patients | 2017 | Moreale, Campanella, Marin, Skrap & Pales | 75 |
| [32] | Long-term cognitive functioning and psychological wellbeing in surgically treated patients with low-grade glioma | 2017 | Campanella, Palese, Del Missier, Moreale, Lus, Shallice, Fabbro & Skrap` | 100 |
| [30] | Internet-based guided self-help for glioma patients with depressive symptoms: a randomised controlled trial | 2018 | Boele, Klein, Verdonck-de Leeuw, Cuijpers, Heimans, Snijders, Vos, Bosma, Tijssen & Reijneveld | 100 |
| [15] | Health-related quality of life in patients with high-risk low-grade glioma (EORTC 22033-26033): a randomised open label phase 3 intergroup study | 2016 | Reijneveld, Taphoorn, Coens, Bromberg, Mason, Hoang-Xuan, Ryan, Hassel, Enting, Brandes, Wick, Chinot, Reni, Kantor, Thiessen, Klein, Verger, Barchers, Hau, Bock, Smits, Galfnopoulos Garlia, Bottomley, Stupp & Baumert | 100 |
| [27] | Psychosocial functioning and quality of life in patients with primary brain tumors | 1996 | Weitzner, Meyers & Byrne | 100 |
| [23] | The Quality of Life of Patients with Malignant Gliomas and Their Caregivers | 2008 | Muñoz, Juarez, Muñoz, Portnow, Fineman, Badie, Mamelak & Ferrel | 75 |
| [22] | The prevalence of altered body image inpatients with primary brain tumours: an understudied population | 2020 | Rowe, Vera, Acquaye, Crandon, Shah, Bryla, Wu, Wall, Siegel, Reyes, Penas-Prado, Leggiero, Cordova, Burton, Antony, Boris, Aboud, Vyas, Mathen, Gilbert, Capmhausen, Mendoza, Armstrong | 75 |
| [25] | Compromised health-related quality of life in patients with low-grade glioma | 2011 | Aaronson, Taphoorn, Heimans, Postma, Gundy, Beute, Slotman & Klein | 100 |
| [21] | Self-Efficacy for coping with cancer in glioma patients measured by the CBI-B | 2019 | Kohlmann, Janko, Ringel & Renovanz | 75 |
| [20] | Health related quality of life in long-term survivors with grade II gliomas: the contribution of disease recurrence and KPS | 2015 | Okita, Narita, Miyahara, Miyaluta, Ohno & Shibiu | 100 |
| [52] | Quality of life in brain tumor patients and their relatives heavily depends on social support factors during the COVID-19 pandemic | 2021 | Troschel, Ahndorf, Wille, Brandt, Jost, Eich, Stummer, Wiewrodt, Jetschke, & Wiewrodt | 75 |
| [31] | Long-term cognitive dysfunction after radiation therapy for gliomas | 2019 | Halbo-Classen, Amidi, Wu, Lukacova, von Oettingen, Gottrup, Zachariae & Høyer | 100 |
| [34] | Long-term outcomes and late adverse effects of a prospective study on proton radiotherapy for patients with low-grade glioma | 2019 | Tibrizi, Yeap, Sherman, Nachtigal, Colvin, Dworkin, Fullerton, Daartz, Royce, Oh, Batchelor, Curry, Loeffler & Shish | 75 |
| [20] | Health-related quality of life in stable, long-term survivors of low-grade glioma | 2015 | Boele, Douw, Reijneveld, Robben, Taphoorn, Aaronson, Heimans & Klein | 75 |
| [24] | The relationship between function, quality of life and coping in patients with low-grade glioma | 2006 | Gustafsson, Edvardsson & Ahlström | 100 |
| [18] | Health-related quality of life and cognitive functioning in long term anaplastic oligodendroglioma & oligoastrocytoma | 2013 | Habets, Taphoorn, Nederend, Klein, Delgadillo, Hoang-Xuan, Bottomley, Allgeier, Seute, Gijtenbeek, Gans, Enting, Tijssen, van den Bent & Reijneveld | 100 |
