# Supplementary material for: Long-term impact of adult WHO grade II or III gliomas on health-related quality of life: A systematic review
Source: Neurooncol Pract. 2021 Nov 10;9(1):3–17. doi: 10.1093/nop/npab062 (PMC8789291; doi:10.1093/nop/npab062)
Supplement: npab062_suppl_Supplementary_Materials_S3 [file npab062_suppl_supplementary_materials_s3.docx]

| Title  Supplementary Material 3 – Excluded Studies | Year | Author | Contacted Author? | Reason for exclusion |
| --- | --- | --- | --- | --- |
| Quality of life and physical limitations in primary brain tumor Patients | 2011 | [Gazzotti](https://pubmed.ncbi.nlm.nih.gov/?term=Gazzotti+MR&cauthor_id=21512831),  [Malheiros](https://pubmed.ncbi.nlm.nih.gov/?term=Malheiros+SM&cauthor_id=21512831), [Batan Alith](https://pubmed.ncbi.nlm.nih.gov/?term=Batan+Alith+M&cauthor_id=21512831),  [Nascimento](https://pubmed.ncbi.nlm.nih.gov/?term=Nascimento+O&cauthor_id=21512831),  [Santoro](https://pubmed.ncbi.nlm.nih.gov/?term=Santoro+IL&cauthor_id=21512831), [Jardim](https://pubmed.ncbi.nlm.nih.gov/?term=Jardim+JR&cauthor_id=21512831),  [Vidotto](https://pubmed.ncbi.nlm.nih.gov/?term=Vidotto+M&cauthor_id=21512831) | Yes | - No mean time since diagnosis included – no reply from corresponding author |
| Quality of life in low-grade glioma patients receiving temozolomide | 2007 | Liu, Solheim, Polley, Lamborn, Page, Fedoroff, Rabbitt, Butowski, Prados, and Chang | Yes | - No mean time since diagnosis included - no reply from corresponding author |
| Relationship between concentrations of IGF-1 and IGFBP-3 and preoperative depression risk and effect of psychological intervention on outcomes of high grade glioma patients with preoperative depression in a 2 year prospective study | 2014 | Wang, Huang, Jiao, Wu, Ouyang, Huang, Liu, Li | Yes | - Clarification needed on tumour pathology and mean time since diagnosis – no reply from corresponding author |
| Development a Model of the Relationship of Psychological Well-Being with Self-Efficacy, Self-Esteem and Psychological Hardiness in Cancer Patients of Benign Brain Tumors | 2016 | Noori, Ahadi, Alzakerini, and Kraskian | Yes | - Mean time since diagnosis not suitable |
| Temozolomide chemotherapy for progressive low-grade glioma: clinical benefits and radiological response | 2003 | [Pace](https://pubmed.ncbi.nlm.nih.gov/?term=Pace+A&cauthor_id=14630675) ,  [Vidiri](https://pubmed.ncbi.nlm.nih.gov/?term=Vidiri+A&cauthor_id=14630675), [Galiè](https://pubmed.ncbi.nlm.nih.gov/?term=Gali%C3%A8+E&cauthor_id=14630675), [Carosi](https://pubmed.ncbi.nlm.nih.gov/?term=Carosi+M&cauthor_id=14630675), [Telera](https://pubmed.ncbi.nlm.nih.gov/?term=Telera+S&cauthor_id=14630675),  [Cianciulli](https://pubmed.ncbi.nlm.nih.gov/?term=Cianciulli+AM&cauthor_id=14630675), [Canalini](https://pubmed.ncbi.nlm.nih.gov/?term=Canalini+P&cauthor_id=14630675),  [Giannarelli](https://pubmed.ncbi.nlm.nih.gov/?term=Giannarelli+D&cauthor_id=14630675), [Jandolo](https://pubmed.ncbi.nlm.nih.gov/?term=Jandolo+B&cauthor_id=14630675), [Carapella](https://pubmed.ncbi.nlm.nih.gov/?term=Carapella+CM&cauthor_id=14630675) | Yes | - Clarification needed on mean time since diagnosis – no reply from corresponding author |
| The prevalence and impact of sleep disturbance on patients with primary brain tumor | 2018 | Garg, Hsu, Salas, Gamaldo, Dzedzic, Lesser, Stowd | Yes | - Clarification was needed on included tumour pathology/grading and mean time since diagnosis |
| Facets and determinants of quality of life in patients with recurrent high grade glioma | 2004 | Giovagnoli Silvani, Colombo, Boiardi | Yes | - Clarification needed of time since diagnosis for tumour group & information on specifically Grade III included tumours – no reply from corresponding author |
| Factors influencing quality of life in patients with benign primary brain tumors: prior to and following surgery | 2012 | Tsay, Chang, Yates, Lin, Liang | Yes | - Needed clarification of tumour pathology and time since diagnosis |
| Health-related quality of life and posttraumatic growth in low-grade gliomas in China: A prospective study | 2018 | Wang, Li, Chen, Fan, Chen, Liu, Chen, Hu | Yes | - Needed clarification of mean time since diagnosis |
| The relationship between function, quality of life and coping in patients with low-grade gliomas | 2006 | Gustafsson, Tanja Edvardsson, Gerd Ahlström | Yes | - Needed clarification of mean time since diagnosis |
| Development of a symptom index for patients with primary brain tumors | 2014 | Lai, Jensen, Beaumont, Abernethy, Jacobsen, Syrjala, Raizer, Cella, | Yes | - Clarification needed on tumour pathology and mean time since diagnosis |
| Health-related quality of life after surgery in supratentorial gliomas. | 2019 | Khatri, Jaiswal, Das, Pandey, Bhaisora, Kumar | Yes | - Upon reply from author, mean time since diagnosis not suitable |
| **Quality of life is independently associated with neurocognitive function in patients with brain tumors: analysis of a prospective clinical trial** | 2021 | Salans, Tibbs, Huynh-Le, Yip, Triangle Karunamuni, Xu, Reyes, Macari, Pan-Weisz, McDonald & Hattangadi-Gluth | No | - Wrong time since diagnosis inferred based on time of treatment and sample of low-grade glioma being < 50% |
| Factors influencing mood disorders and health related quality of life in adults with glioma: A longitudinal study | 2021 | Leonetti, Puglisi, Rossi, Vigano, Nibali. Gay, Sciortino, Howells, Fornia, Riva, Cerri & Bello | No | - Wrong time since diagnosis inferred based on standardised timeline of treatment. |
